# Supplementary material for: AffyMAPSDetector: a software tool to characterize Affymetrix GeneChip™ expression arrays with respect to SNPs
Source: BMC Bioinformatics. 2007 Jul 30;8:276. doi: 10.1186/1471-2105-8-276 (PMC1959249; doi:10.1186/1471-2105-8-276)
Supplement: Additional file 13 — AffyMAPSDetector v1 source code. AffyMAPSDetector source code. [file 1471-2105-8-276-S13.zip › AffyMAPSDetector_v1_src/doc/index-files/index-13.html]

N-Index


|  |  |  |  |
| --- | --- | --- | --- |
| **Overview** \| Package \| Class \| Use \| **Tree** \| **Deprecated** \| **Index** \| **Help** | | |  |
| **PREV LETTER**   **NEXT LETTER** | **FRAMES**    **NO FRAMES**     **All Classes** |  |  |


A B C D E F G H I J L M N O P R S T V W X Y 

---


## **N**

**NATURAL\_LOG\_2** - Static variable in class affychip.snpdetector.Constant: **NEXT\_I18N** - Static variable in class jwf.Wizard: **NO\_LOCUS\_LINK\_FILENAME** - Static variable in class affychip.snpdetector.Constant: **NO\_SNPS\_FILENAME** - Static variable in class affychip.snpdetector.Constant: **NullWizardPanel** - class jwf.NullWizardPanel.: An implementation of the base class used for implementing a panel that is displayed in a Wizard. **NullWizardPanel()** - Constructor for class jwf.NullWizardPanel: A default constructor. **ncbiGeneAccessionNoSearchUrl** - Static variable in class affychip.snpdetector.model.SnpDetector: **ncbiNucleotideDbUrl** - Static variable in class affychip.snpdetector.model.SnpDetector: **ncbiRefSnpClusterUrl** - Static variable in class affychip.snpdetector.model.SnpDetector: **ncbiSnpDbUrl** - Static variable in class affychip.snpdetector.model.SnpDetector: **next()** - Method in class affychip.snpdetector.ui.StepOnePanel: Get the next panel to go to. **next()** - Method in class affychip.snpdetector.ui.StepThreePanel: **next()** - Method in class affychip.snpdetector.ui.StepTwoPanel: **next()** - Method in class jwf.NullWizardPanel: Get the next panel to go to. **next()** - Method in class jwf.WizardPanel: Get the next panel to go to.

---


|  |  |  |  |
| --- | --- | --- | --- |
| **Overview** \| Package \| Class \| Use \| **Tree** \| **Deprecated** \| **Index** \| **Help** | | |  |
| **PREV LETTER**   **NEXT LETTER** | **FRAMES**    **NO FRAMES**     **All Classes** |  |  |


A B C D E F G H I J L M N O P R S T V W X Y 

---
